# Supplementary material for: Anti-Toxin Responses to Natural Enterotoxigenic Escherichia coli (ETEC) Infection in Adults and Children in Bangladesh
Source: Microorganisms. 2023 Oct 9;11(10):2524. doi: 10.3390/microorganisms11102524 (PMC10609113; doi:10.3390/microorganisms11102524)
Supplement: Supplementary file 1 [file microorganisms-11-02524-s001.zip › microorganisms-2609858-supplementary.pdf]

Supplementary Material

Table S1

		anti-LTB IgG			anti-LTB IgA			anti-LTA IgG			total responders		
		LT+/ST-	LT+/ST+	ST+	LT+/ST-	LT+/ST+	ST+	LT+/ST-	LT+/ST+	ST+	LT+/ST-	LT+/ST+	ST+
Children	2 fold	3 (9%)	5 (15%)	2(17%)	5(15%)	2(6%)	4(27%)	2(6%)	4(12%)	1(8%)	7(21%)	6(18%)	5(42%)
	4 fold	1 (3%)	3 (9%)	1(8%)	1(3%)	0(0%)	1(7%)	1 (3%)	3 (9%)	0(0%)	2(6%)	3(9%)	2(17%)
Adults	2 fold	1(4%)	4(14%)	1(7%)	1(4%)	2(8%)	0(0%)	1(4%)	4(14%)	1(7%)	1(4%)	4(14%)	2(14%)
	4 fold	1(4%)	2(7%)	0(0%)	0(0%)	1(4%)	0(0%)	1(4%)	2(7%)	0(0%)	1(4%)	2(7%)	0(0%)

Table S1: Anti-LTB IgG/IgA and anti-LTA IgG responder rates for ETEC infection grouped based on the presence of the LT and ST genes. Number of patients and their percentage in corresponding population with 2- or 4- fold increase in plasma IgG or IgA titers at any sampling day compared to day 2 (or IgG) or day 30 (for IgA) are given for LTB and LTA separately, or for all subjects with 2- or 4-fold increase for any of the antigen or antibody isotype tested (total responders).

Table S2

		ELISA (end point titers)					
Subject ID	Subject type	LTB IgG			CTB IgG		
		D2	D7	D30	D2	D7	D30
D0110018	LT/STh, adult	866	1133	1866	67	76	131
D0110020	LT/STh, adult	2389	2601	2202	57	52	30
D0110022	LT/STh, adult	2303	28677	10171	127	3.051	498
D0110016	LT/STp, adult	473	416	524	48	507	588
D0110024	LT, adult	583	4910	3292	29	1.397	1.168
M0210073	STh, adult	243	299	822	70	71	133
M0220023	LT/STh, 9 months	1342	10007	5561	81	485	197
M0220063	LT/STh, 3 years	691	455	1522	123	1.504	607
M0220057	LT/STh, 3 years	1080	840	987	114	94	190
M0220005	LT/STp, 1 year	699	1089	1123	170	97	106
M0220062	LT/STp, 2 years	1841	19657	13957	235	3.952	3.146
M0220133	LT/STp, 2 years	944	904	746	58	87	85
M0220061	LT, 1 year	1027	2623	6227	96	562	1.731
M0220064	LT, 1 year	1670	2165	1853	148	160	148
M0220086	LT, 3 years	22690	16360	6890	4.762	4.280	2.256
M0220096	LT, 2 years	793	1463	908	141	206	148
M0220104	LT, 2 years	11782	24777	15389	1.725	2.766	2.102

		ELISA (end point titers)					
Subject ID	Subject type	LTB IgG			CTB IgG		
		D2	D7	D30	D2	D7	D30
M0220172	LT, 1 year	2549	4582	1636	774	898	578
M0220056	STh, 3 years	2745	2740	2217	434	419	393
M0220077	STh, 4 years	3082	823	813	202	143	168
M0220157	STp, 4 years	8410	8421	5135	262	259	301
M0250225	healthy adult	2582			141		
M0250215	healthy adult	1434			118		

Table S2: Anti-LTB IgG and anti-CTB IgG end point titers for selected patients with ETEC infection or healthy adults. Subject ID, Subject type including age of patient and type of ETEC infection based on toxin expression and corresponding plasma anti-LTB anti-CTB IgG end point titers at day 2, day 7 and day 30 after onset of symptoms for selected patients with ETEC infection or at on time point for healthy adults.
